# Identification of Exercise-Related Signature Genes Potentially Associated with Cocaine Addiction by Integrating Bioinformatics and Mendelian Randomization Analysis

**DOI:** 10.3390/genes16121414

**Published:** 2025-11-27

**Authors:** Jinke He, Xiaoyu Deng, Yuxuan Deng, Xiao Huang

**Affiliations:** 1 Hunan Provincial Key Laboratory of Dong Medicine, Biomedical Research Institute, Hunan University of Medicine, Huaihua 418000, China; 2 School of Basic Medical Sciences, Xinjiang Second Medical College, Kelamayi 834000, China; 3 School of Basic Medical Sciences, Hunan University of Medicine, Huaihua 418000, China; 4 School of Clinical Medical, Hunan University of Medicine, Huaihua 418000, China

**Keywords:** cocaine addiction, exercise, signature genes, bioinformatics, Mendelian randomization, immune infiltration

## Abstract

**Background**: Exercise is a promising non-pharmacological intervention for cocaine addiction but molecular mechanisms of exercise-related genes in addiction remain unclear. This study aimed to identify exercise-related signature genes for cocaine addiction and to assess the potential causal relationship between exercise and cocaine addiction using two-sample Mendelian randomization (MR) analysis. **Methods**: Midbrain transcriptomic data were analyzed for differentially expressed genes (DEGs) and intersected with exercise-related genes. Functional enrichment, protein-protein interaction (PPI) and immune infiltration analyses explored their roles while signature genes were screened via LASSO/Random Forest and validated by ROC curves. GSEA explored pathways and MR confirmed exercise’s causal effect. **Results**: A total of 244 DEGs were identified, including 27 exercise-related, and six signature genes (CALM3, CCL2, CD44, CLIC1, JUN, VCAM1) showed AUC values between 0.714 and 0.868 in distinguishing cocaine-addicted individuals from controls. Functional analyses revealed enrichment in immune-inflammatory pathways, metabolic processes and neuro-immune interactions and immune infiltration analysis showed cocaine addicts had elevated pro-inflammatory cells, reduced regulatory cells and signature genes correlated with immune dysregulations. MR analysis suggested a statistically significant protective association between genetically proxied higher levels of exercise and cocaine addiction risk (*p* < 0.05). **Conclusions**: These six genes may be potential biomarkers and therapeutic targets, and exercise may protect against cocaine addiction by regulating immune-inflammatory responses, metabolic pathways and neuroplasticity, although further validation in larger, independent cohorts and experimental models is required.

## 1. Introduction

Drug addiction remains a devastating public health crisis globally, characterized by compulsive drug-seeking behavior, high relapse rates, and profound neurobiological dysregulation [1,2]. Among addictive substances, cocaine stands out as a highly addictive drug widely abused for its potent excitatory effects on the central nervous system. Since 1985, it has emerged as one of the world’s most prevalent drugs, particularly in the Americas and Europe [3]. Its pathogenesis involves complex interactions across neural circuits (such as reward pathways mediated by dopamine), immune activation, and transcriptional reprogramming in key brain regions such as the midbrain, a critical hub for addiction-related neuroplasticity [4,5]. Despite decades of research, the molecular mechanisms underlying cocaine addiction, particularly the genetic and regulatory networks driving susceptibility and persistence, remain incompletely understood [6]. This knowledge gap hinders the development of targeted interventions.

Exercise has emerged as a promising non-pharmacological strategy to mitigate addiction-related behaviors. However, the interplay between exercise and drug addiction constitutes a complex investigative landscape, spanning molecular, immunological, and epigenetic dimensions [6,7,8]. Exercise-modulated signature genes, whose expression is reprogrammed by physical activity, are postulated to mediate protective effects against drug addiction, participating in biological processes such as neuroadaptive remodeling, immune microenvironment tuning, and metabolic reprogramming [9,10,11]. Preclinical and clinical evidence has demonstrated exercise’s efficacy in mitigating drug-seeking behaviors, enhancing impulse control, and normalizing neurochemical dysregulation [12,13]. However, the molecular circuitry linking exercise-responsive genes to the transcriptional and immunological hallmarks of cocaine addiction remains incompletely characterized [14]. Existing studies have largely focused on behavioral endpoints or single-pathway analyses, lacking systems-level integration of transcriptomic data, immune dynamics, and causal inference to pinpoint core regulatory genes [15,16]. Deciphering this multiscale interaction is pivotal for elucidating exercise’s protective mechanisms and devising targeted interventions.

Accumulating clinical and preclinical evidence indicates that multiple forms of physical activity, including aerobic training, resistance exercise and mind–body modalities, can reduce craving, improve mood symptoms and enhance abstinence rates in individuals with substance use disorders [17,18]. Recent meta-analyses and randomized trials commonly categorize exercise prescriptions by type, intensity, frequency and session duration, and report broadly consistent benefits of regular moderate-to-vigorous aerobic exercise on craving and relapse risk, with additional, though sometimes less consistent, effects of resistance or combined regimens [18,19]. In the present study, we therefore focused on genes that have been repeatedly implicated as exercise-responsive across different modalities and intensities, treating them as shared molecular signatures of habitual physical activity rather than markers of a single, highly specific exercise protocol.

In recent years, bioinformatics tools such as high-throughput sequencing and microarray technology have advanced continuously and been widely applied in molecular biology research [20]. This progress enables efficient detection of biological information including differentially expressed genes (DEGs) and their functional networks, thus providing robust support for exploring the molecular mechanisms of addiction [3]. In parallel, Mendelian randomization (MR) uses genetic variants as instrumental variables to strengthen causal inference between exposures (such as exercise) and outcomes (such as addiction), helping to disentangle complex associations in addiction research [21]. For MR to be valid, the selected genetic instruments are expected to be robustly associated with the exposure, independent of major confounders and to affect the outcome primarily through the exposure, which constitute its core assumptions [21]. Together, these bioinformatics and MR approaches provide complementary frameworks for characterizing high-throughput omics datasets [22], identifying addiction-related signature genes and evaluating the potential causal relationship between exercise and cocaine addiction.

In this study, we aimed to identify exercise-related signature genes associated with cocaine addiction through a multi-step bioinformatics pipeline, including DEG analysis, protein-protein interaction (PPI) networking, functional enrichment, immune infiltration profiling, and machine learning-based gene prioritization. Furthermore, we employed MR to assess the potential causal relationship between exercise and cocaine addiction risk. This study seeks to uncover novel molecular targets underlying exercise-mediated protection against cocaine addiction, providing a theoretical basis for developing exercise-based therapeutic strategies.

## 2. Materials and Methods

### 2.1. Data Source

The gene expression datasets were retrieved from the Gene Expression Omnibus (GEO) database (Accession: GSE54839, URL: https://www.ncbi.nlm.nih.gov/geo/, accessed on 2 July 2025). Using the search terms ‘cocaine’ AND ‘addiction’, we identified all cocaine addiction-related datasets and applied the following inclusion criteria: (1) human case-control studies, (2) chronic cocaine abusers, and (3) microarray-based profiling. The selected GSE54839 dataset comprises transcriptomic profiles of the midbrain from 10 chronic cocaine abusers and 10 matched drug-free controls. Data generation was performed on the Illumina HumanHT-12 V3.0 expression beadchip platform (GPL6947).

### 2.2. Identification of Exercise-Related Differentially Expressed Genes

Initially, the GSE54839 dataset was retrieved and processed in R. Differentially expression genes (DEGs) between the cocaine addiction group and the control group were identified using the ‘limma’ package, with a significance threshold of *p* < 0.05 and fold-change ≥1.3. DEGs were visualized as a heatmap and volcano plot using the ‘pheatmap’ and ‘ggplot2’ packages, respectively. Subsequently, exercise-related genes were queried from the GeneCards database (https://www.genecards.org, accessed on 2 July 2025) using the keywords aerobic exercise, anaerobic exercise and exercise. The intersection between DEGs and exercise-related genes was extracted to derive differentially expressed exercise-related genes. Finally, principal component analysis (PCA) was performed on these genes to evaluate the reproducibility of sample clustering within groups.

Given the relatively small sample size (n = 10 per group), we additionally performed a simulation-based post hoc power analysis calibrated to the empirical gene-wise variance estimates from the limma model. Using the 90th percentile of the residual standard deviation distribution (σ ≈ 0.21 on the log_2_ scale) as a conservative noise estimate, two-sample *t*-test simulations (n = 10 vs. 10, α = 0.05, 30,000 iterations) yielded approximately 89% power to detect genes with log_2_ fold changes of 0.3 (~1.23-fold), 99% power for log_2_ fold changes of 0.4 (~1.32-fold), and ≥99% power for log_2_ fold changes ≥0.5 (~1.41-fold, Supplementary Table S4).

### 2.3. Protein Interaction Networks and Principal Component Analysis of Exercise-Related Differential Genes

The STRING database (https://string-db.org, accessed on 6 July 2025) was employed to predict molecular interactions and protein-protein interaction (PPI) networks for exercise-related differentially expressed genes (DEGs). Hub genes were subsequently prioritized from these DEGs using Cytoscape’s degree centrality algorithm. Pairwise correlations among prioritized genes were finally analyzed using the ‘corrplot’ R package (version 0.95).

### 2.4. Functional and Pathway Enrichment Analysis of Exercise-Related DEGs

We performed functional enrichment analysis of exercise-related differentially expressed genes (DEGs) using the KEGG database (https://www.kegg.jp, accessed on 6 July 2025). The ‘ClusterProfiler’ R package was employed for Gene Ontology (GO) and Kyoto Encyclopedia of Genes and Genomes (KEGG) pathway enrichment analyses, with statistical significance defined as *p* < 0.05. The GO analysis encompassed three ontological categories: biological processes (BP), cellular components (CC), and molecular functions (MF). These categories were examined to identify biological processes associated with the DEGs, while KEGG analysis elucidated potential signaling pathways involving these genes.

### 2.5. Immunoinfiltration Analysis of DEGs

Differential gene expression data were analyzed using the R package “CIBERSORT” to determine the distribution of 22 immune cell types in the brain tissues of cocaine addicts. Additionally, we analyzed the distribution of immune cell types associated with exercise-related differential genes. Correlation and infiltration analyses were also performed on immunological parameters.

### 2.6. Identification of Signature Genes from Exercise-Related DEGs

Signature genes were screened from exercise-related differentially expressed genes (DEGs) in cocaine-addicted individuals using two machine learning algorithms: Least Absolute Shrinkage and Selection Operator (LASSO) regression and Random Forest (RF). The final signature genes were determined by intersecting the candidate genes identified from both methods. When assessing high-dimensional data, LASSO outperformed classical regression techniques [23]. The “glmnet” R package was employed for LASSO analysis, with the penalty parameter established via 10-fold cross-validation. The Random Forest algorithm, typically applied to large-scale datasets with high dimensionality, identified the optimal number of variables by computing the mean decrease in accuracy of candidate genes. Signature relevance of key candidate genes was subsequently evaluated based on this metric [24]. The diagnostic efficacy of these signature genes was assessed using the Area Under the Receiver Operating Characteristic Curve (AUC-ROC), where an AUC value ≥0.7 indicated robust diagnostic performance.

### 2.7. GSEA Enrichment Analysis of Signature Genes

To investigate associations between signature genes and signaling pathways, cocaine-addicted subjects were stratified into subgroups based on the median expression of signature genes. Gene Set Enrichment Analysis (GSEA) was performed on these subgroups, with adjusted *p*-values < 0.05 considered statistically significant. The diagnostic performance of individual genes was evaluated using the Area Under the Receiver Operating Characteristic Curve (AUC-ROC).

### 2.8. Mendelian Randomization Analysis

This study employed a two-sample Mendelian randomization (MR) approach to investigate the potential causal association between exercise and cocaine addiction risk. Summary-level genome-wide association study (GWAS) data for exercise-related traits (exposure; ukb-B-4000) and cocaine addiction (outcome; ukb-d-20552_2) were obtained from large publicly available resources, and exercise-associated single-nucleotide polymorphisms (SNPs) reaching a suggestive significance threshold (*p* < 1 × 10^−5^) were extracted as instrumental variables (IVs), then pruned for linkage disequilibrium (r^2^ < 0.001 within a 10,000-kb window) and required to have F-statistics ≥10 (Supplementary Table S5) to minimize weak-instrument bias.

Heterogeneity and potential pleiotropy were evaluated using Cochran’s Q statistic, the MR-Egger intercept test and the MR-PRESSO global test, with inverse-variance weighted (IVW) estimates complemented by MR-Egger, weighted median, weighted mode and simple mode methods implemented in the TwoSampleMR R package (v0.6.17).

### 2.9. Statistical Analysis

**Figure 1:**
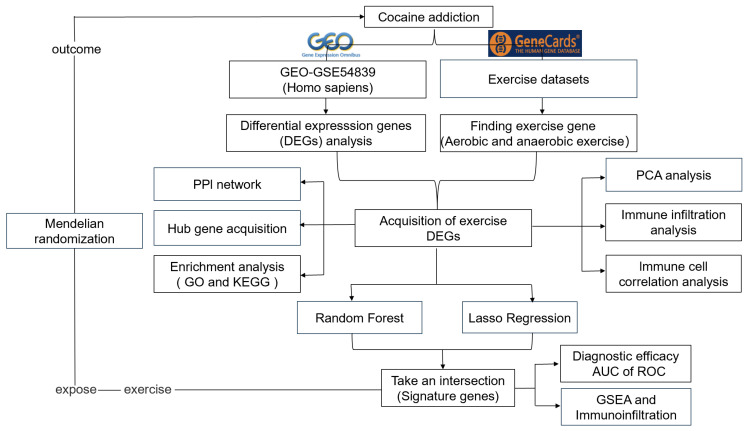
Flow chart of this study.

Statistical analyses were performed using R software (version 4.3.3). A predefined significance threshold of 0.05 was applied to determine statistically significant differences. The flowchart of the study design is presented in Figure 1.

## 3. Results

### 3.1. Identification of DEGs Between the Cocaine Addiction and Control Group

**Figure 2:**
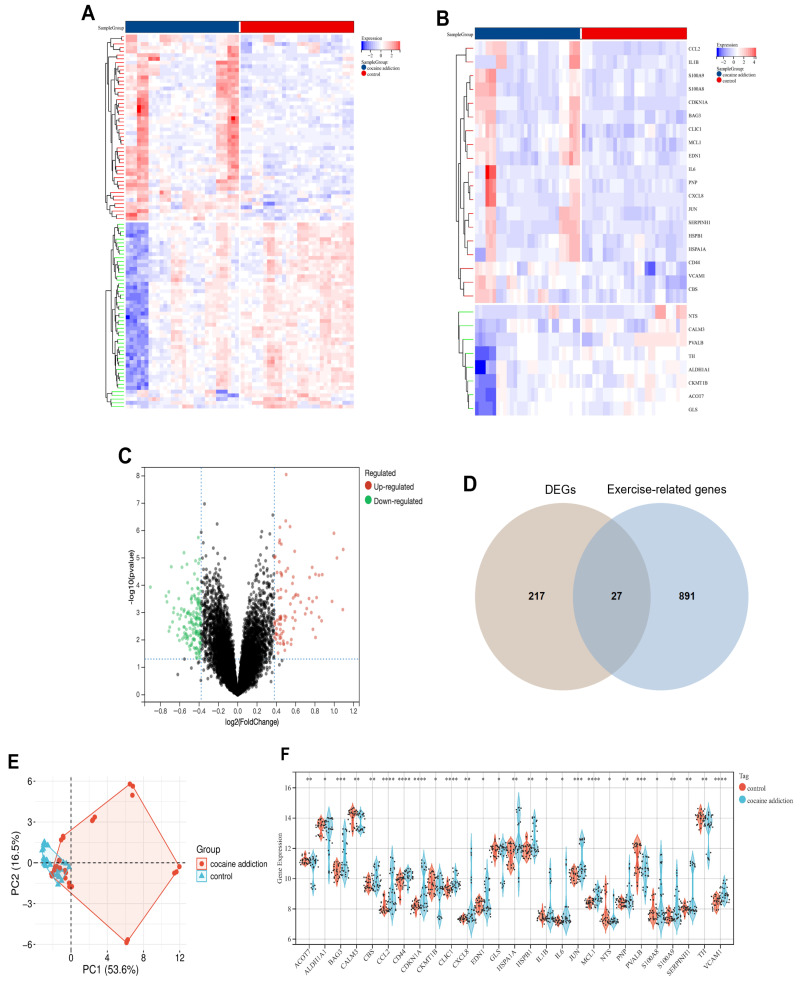
Identification of DEGs in Cocaine Addiction. (**A**) Heatmap of DEGs in cocaine addiction. (**B**) Heatmap of exercise-related DEGs in cocaine addiction. (**C**) Volcano plot of DEGs in cocaine addiction. (**D**) Venn diagram of intersection between cocaine addiction DEGs and exercise-related genes. (**E**) Principal component analysis (PCA) of exercise-related DEGs in cocaine addiction. (**F**) Histogram of expression levels for 27 exercise-related DEGs in cocaine addiction. *: *p* < 0.05; **: *p* < 0.01; ***: *p* < 0.001; ****: *p* < 0.0001.

Analysis of the GSE54839 dataset (cocaine-addicted vs. control groups) using the R package “limma” identified 244 differentially expressed genes (DEGs) (Figure 2A,C), comprising 92 upregulated and 152 downregulated genes. We queried the GeneCards database for 918 genes associated with aerobic and anaerobic exercise. Twenty-seven exercise-related DEGs were obtained by intersecting the DEGs with identified exercise genes (Figure 2B,D,F). Table 1 shows the top five upregulated genes (*CCL2*, *CDKN1A*, *HSPA1A*, *SERPINH1*, *JUN*) and top five downregulated genes (*PVALB*, *TH*, *CKMT1B*, *ALDH1A1*, *GLS*). To assess intra-group reproducibility and interpret gene expression variance, an additional principal component analysis (PCA) was performed. The results in Figure 2E demonstrate high reproducibility of data derived from exercise-related DEGs.

### 3.2. PPI Network Construction, Correlation and Functional Enrichment Analysis of Exercise-Related DEGs

**Figure 3:**
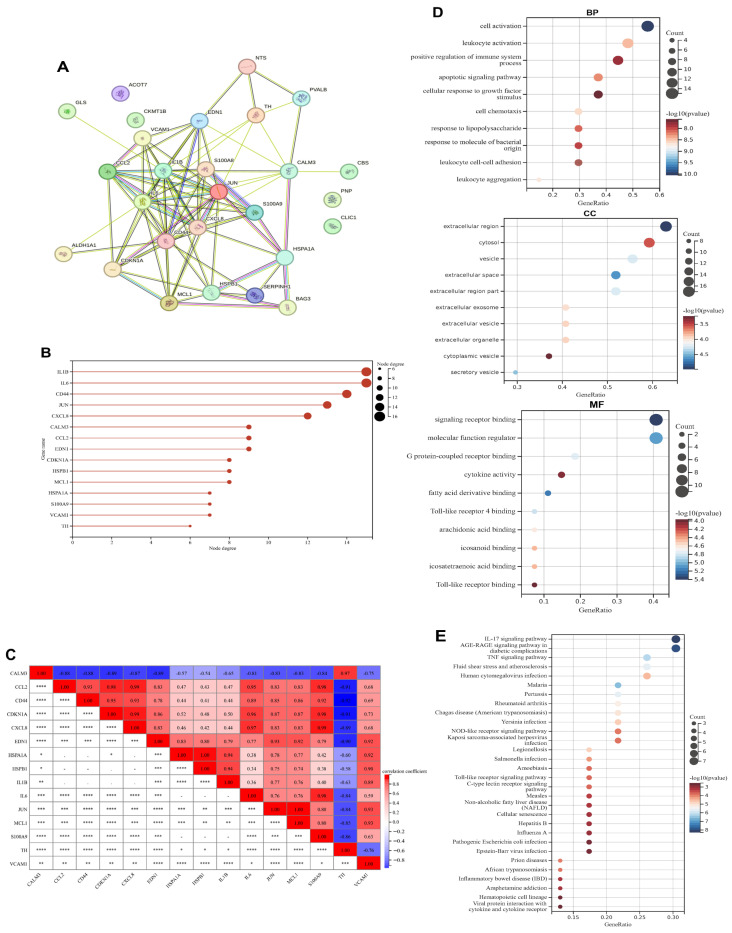
PPI Network, Correlation and Enrichment Analyses of Exercise-Related DEGs in Cocaine Addiction. (**A**) Protein-protein interaction (PPI) network of exercise-related DEGs. (**B**) Top 15 hub genes in the PPI network. (**C**) Pearson correlation analysis of 15 hub genes. (**D**) Gene Ontology (GO) enrichment analysis of exercise-related DEGs; (**E**) KEGG pathway enrichment analysis of exercise-related DEGs. *: *p* < 0.05; **: *p* < 0.01; ***: *p* < 0.001; ****: *p* < 0.0001.

To investigate the functional roles and interactions of these genes, we constructed a protein-protein interaction (PPI) network for hub genes using the STRING online tool (Figure 3A). The top 15 hub genes were identified via Cytoscape software (3.10.4) (Figure 3B), comprising *IL1B*, *IL6*, *CD44*, *JUN*, *CXCL8*, *CALM3*, *CCL2*, *EDN1*, *CDKN1A*, *HSPB1*, *MCL1*, *HSPA1A*, *S100A9*, *VCAM1*, and *TH*. Furthermore, Pearson correlation analysis was performed. As demonstrated in Figure 3C, *CCL2* exhibited strong positive correlations with *CD44*, *CDKN1A*, and *CXCL8*. CD44 showed significant positive correlations with *CDKN1A* and *CXCL8*, *CDKN1A* displayed robust positive correlations with *CXCL8* and *JUN*, *JUN* demonstrated prominent positive correlation with *MCL1*, *EDN1* correlated positively with HSPA1A and *HSPB1*. Conversely, TH manifested strong negative correlations with CCL2, CD44, *CDKN1A*, and *CXCL8*, while *IL6* and *S100A9* were also negatively correlated with *TH*.

Significant enrichment of extracellular components and immune-related pathways was observed in the GO analysis. Cellular Component (CC) terms were predominantly associated with the extracellular space and vesicular structures, including extracellular region, exosome, secretory vesicle, and cytoplasmic vesicle. Biological Process (BP) analysis revealed profound involvement of immune activation mechanisms, with top enriched terms encompassing leukocyte activation, positive regulation of immune system processes, and response to bacterial molecules (e.g., lipopolysaccharide). Molecular Function (MF) highlighted ligand-receptor interactions, particularly signaling receptor binding, Toll-like receptor binding (including TLR4), and lipid mediator binding (e.g., icosanoid, arachidonic acid) (Figure 3D).

Robust enrichment of inflammation and infection-related pathways was identified through KEGG analysis. The most significantly enriched terms centered on immune signaling cascades, including the IL-17 signaling pathway, TNF signaling pathway, and Toll-like receptor signaling pathway. Pathogen-specific responses were prominently featured, with high enrichment for Human cytomegalovirus infection, Salmonella infection, Malaria, and Epstein-Barr virus infection. Chronic inflammatory diseases such as Rheumatoid arthritis, Inflammatory bowel disease (IBD), and Non-alcoholic fatty liver disease (NAFLD) were also significantly represented. Notably, neuro-immune interactions were implicated through the enrichment of Amphetamine addiction pathway and AGE-RAGE signaling in diabetic complications (Figure 3E).

### 3.3. Immunological Characterization of Cocaine Addiction and Exercise-Related DEGs

**Figure 4:**
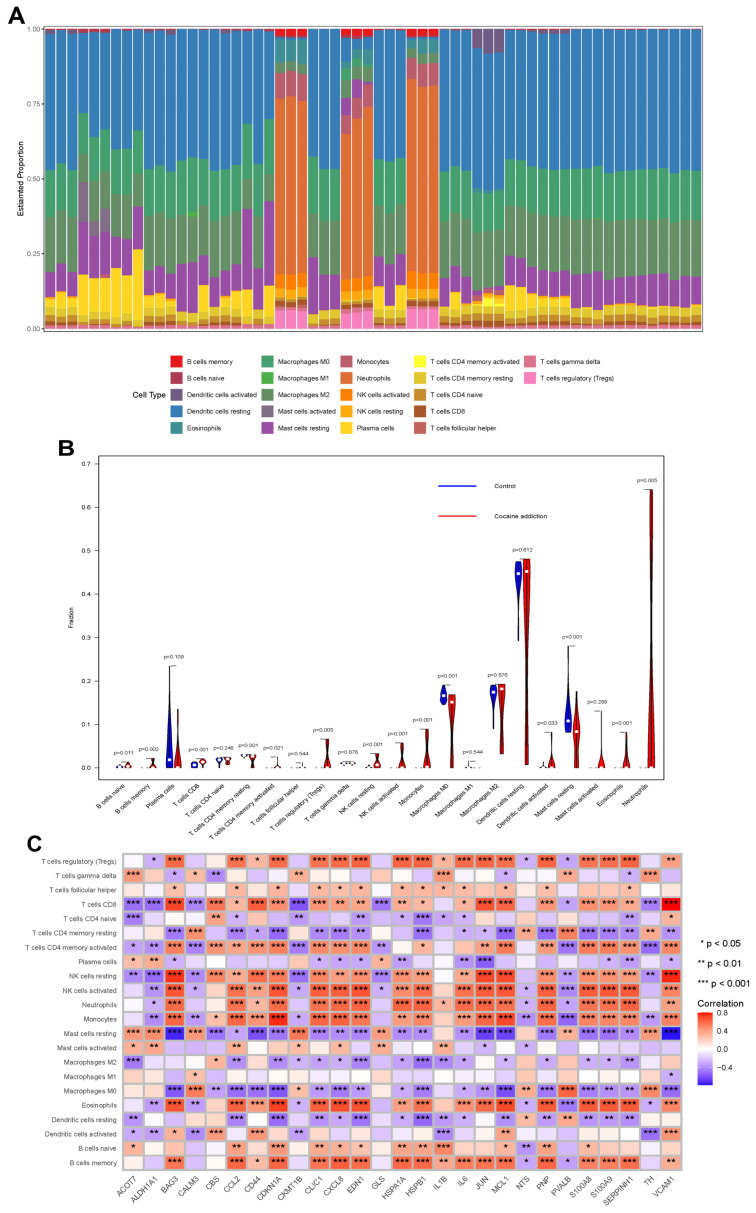
Immunological characterization of cocaine addiction DEGs and exercise-related DEGs. (**A**) Relative abundance of 22 immune cell subtypes in cocaine-addicted DEGs. (**B**) Differential immune cell infiltration between cocaine-addicted and control DEG cohorts. (**C**) Immune cell subpopulations associated with 27 exercise-modulated DEGs in cocaine addiction.

CIBERSORT analysis quantified immune cell proportions in cocaine-addicted individuals (Figure 4A). Memory B cells constituted the dominant subset (75% relative abundance), with M0 macrophages (68%) and monocytes (52%) representing secondary major populations. Notably, immunosuppressive cell types were markedly reduced; regulatory T cells (Tregs) and resting dendritic cells each accounted for less than 10% of total infiltrate. This skewed profile, characterized by elevated pro-inflammatory effectors such as M0/M1 macrophages and neutrophils concurrent with activated adaptive immune cells including CD4^+^ memory T cells and T follicular helper cells, indicates a chronically inflamed microenvironment exhibiting compromised immunoregulatory function.

Comparative analysis revealed significant immune dysregulation in cocaine addiction versus controls (Figure 4B). The cocaine cohort demonstrated substantial expansion of M0 macrophages (*p* < 0.001), neutrophils (*p* = 0.002), and memory B cells (*p* = 0.004). Conversely, profound depletion was observed in regulatory T cells (Tregs, *p* < 0.001) and resting dendritic cells (*p* = 0.007). Key fold-change measurements showed M0 macrophages with a 2.2-fold increase and Tregs with a 3.1-fold decrease. This bidirectional dysregulation (*p* < 0.05 for 15/22 cell types) establishes a pathological immune milieu conducive to sustained neuroinflammation. For exercise-related DEGs (n = 27), immune infiltration analysis (Figure 4C) revealed positive correlations between key DEGs and proinflammatory subsets (e.g., activated T cells, neutrophils) alongside negative associations with resting immune populations (e.g., mast cells) (*p* < 0.001). These patterns imply exercise-driven immune modulation, contrasting with the pathological immune skew in cocaine addiction.

### 3.4. Identification and Characterization of Exercise-Related Signature Genes

**Figure 5:**
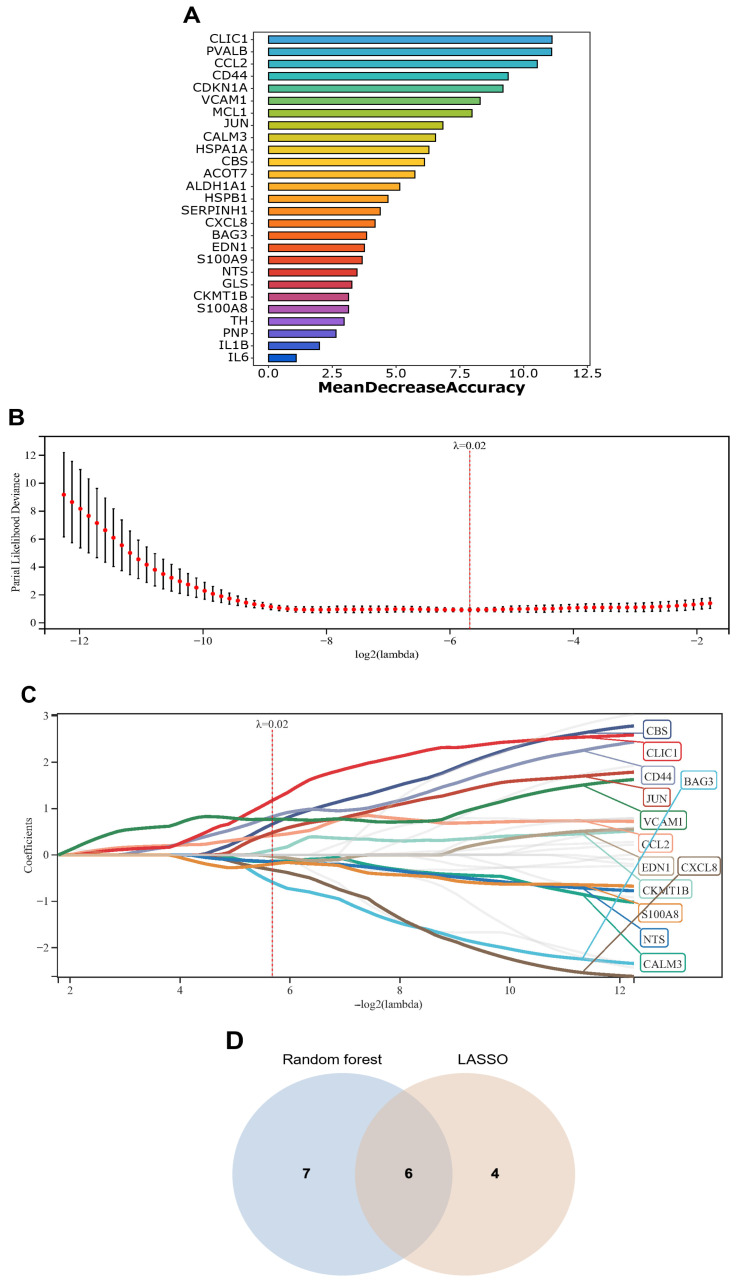
Machine algorithm of exercise-related signature genes. (**A**) Random Forest-based feature importance ranking of exercise-related signature genes. (**B**) LASSO regression-derived lambda selection plot using partial likelihood deviance. (**C**) LASSO regression coefficient profiles of exercise-related signature genes across lambda gradients. (**D**) Venn diagram depicting the intersection of genes identified by LASSO regression and random forest algorithm.

To enhance the consistency and reliability of signature genes selection, we employed two machine learning algorithms: random forest (RF) and least absolute shrinkage and selection operator (LASSO) regression to identify signature genes from 27 exercise-related DEGs in the cocaine addiction context, and determined their intersection (Figure 5A). LASSO analysis identified 13 signature genes, while RF analysis selected 10 genes with Mean Decrease Accuracy (MDA) >6 (Figure 5B,C). Through cross-validation of both algorithms, we identified six consensus signature genes *CALM3*, *CCL2*, *CD44*, *CLIC1*, *JUN*, and *VCAM1* (Figure 5D).

### 3.5. The Value of Exercise-Related Signature Genes in the Diagnosis of Cocaine Addiction

**Figure 6:**
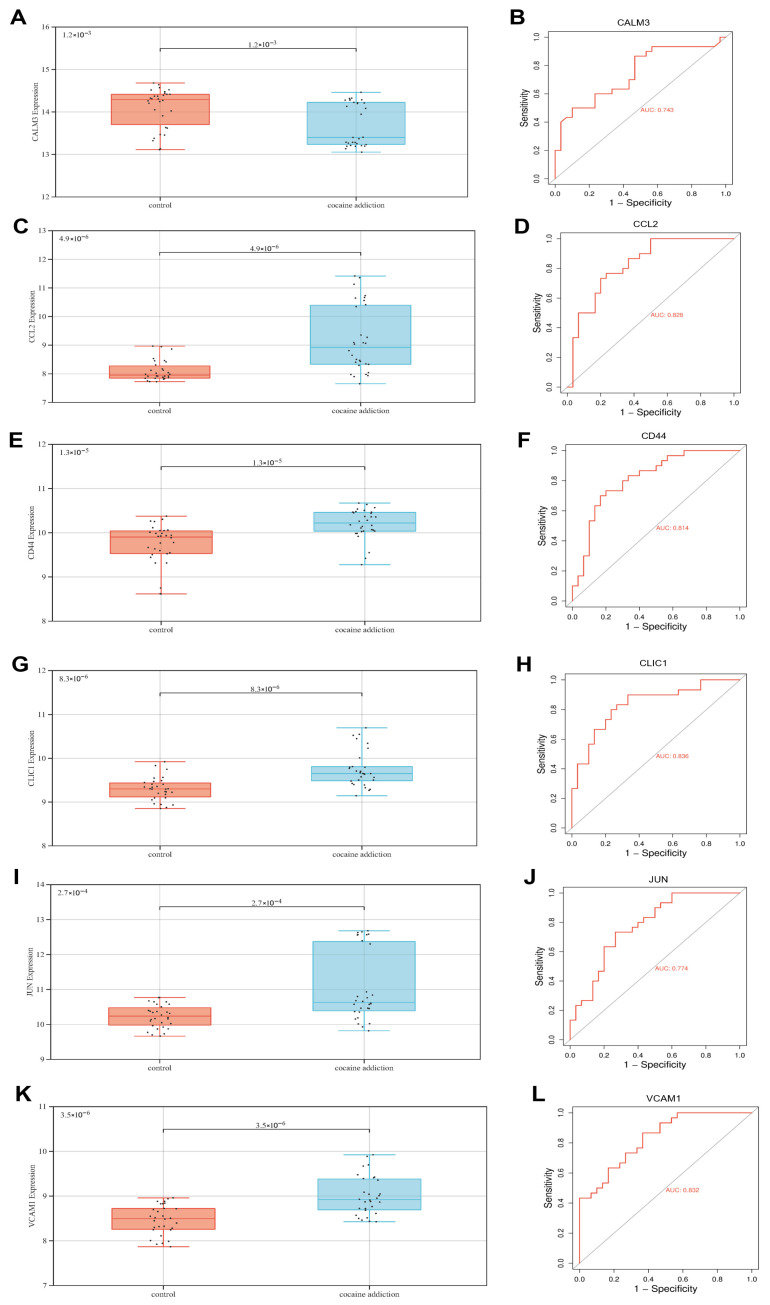
The diagnostic efficacy of exercise-related signature genes in cocaine addiction was analyzed by box plot and ROC. (**A**,**C**,**E**,**G**,**I**,**K**) Expression of exercise-related signature genes in cocaine addiction group and control group. (**B**,**D**,**F**,**H**,**J**,**L**) ROC represents the diagnostic performance of exercise-related signature genes.

To evaluate the diagnostic value of six exercise-related signature genes in cocaine addiction, we analyzed their expression differences via boxplots and assessed diagnostic performance using receiver operating characteristic (ROC) curves (Figure 6A–L). *CALM3* and *JUN* were downregulated in the cocaine addiction group compared to controls (*p* < 0.05), whereas *CCL2*, *CD44*, *CLIC1*, and *VCAM1* were upregulated (*p* < 0.05). ROC curve analysis further indicated moderate discriminative performance, with area under the curve (AUC) values ranging from 0.714 (*CALM3*) to 0.868 (*CCL2*). Notably, *CCL2* (AUC = 0.868), *CD44* (AUC = 0.814), *CLIC1* (AUC = 0.816), and *VCAM1* (AUC = 0.828) exhibited AUCs > 0.8, but these estimates were derived from the same dataset used for feature selection and may reflect optimistic in-sample performance, and thus require confirmation in larger, independent cohorts before any clinical application can be considered.

### 3.6. Functional Analysis of Exercise-Related Signature Genes and Correlation Analysis of Immune Infiltration

**Figure 7:**
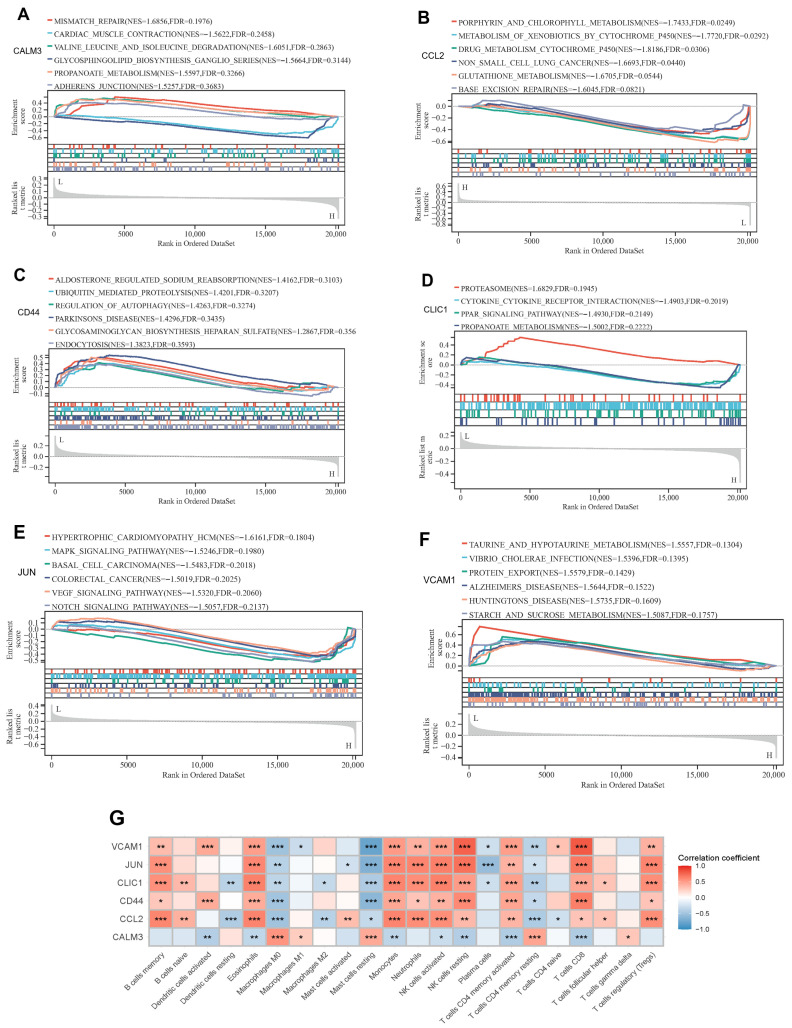
GSEA of exercise-related signature genes and immune cell infiltration. (**A**–**F**) GSEA of exercise-related signature genes. (**G**) Correlation between exercise-related signature genes and immune cell infiltration. *: *p* < 0.05; **: *p* < 0.01; ***: *p* < 0.001.

To elucidate the functional mechanisms and immunomodulatory roles of the six exercise-related signature genes (*CALM3*, *CCL2*, *CD44*, *CLIC1*, *JUN*, *VCAM1*) in cocaine addiction, gene set enrichment analysis (GSEA) and immune cell infiltration correlation analysis were performed. GSEA (Figure 7A–F) revealed that these genes are enriched in diverse functional pathways, *CALM3* participates in asparagine metabolism, adherens junction formation, and branched—chain amino acid degradation, indicating its involvement in metabolic reprogramming and cell-cell adhesion. *CCL2* is enriched in porphyrin/chlorophyll metabolism, glutathione biosynthesis, and cytokine signaling, highlighting its central role in redox homeostasis and immune modulation. *CD44* is associated with aldosterone-regulated sodium reabsorption, autophagy regulation, and glycosaminoglycan biosynthesis, suggesting its contribution to cellular homeostasis and extracellular matrix interactions. *CLIC1* focuses on proteasomal degradation, pyruvate metabolism, and propanoate biosynthesis, underscoring its function in metabolic rewiring. *JUN* is enriched in hypertrophic cardiomyopathy, MAPK signaling, and VEGF pathways, indicating its integration of cellular stress responses and growth signaling. *VCAM1* is involved in taurine metabolism, xenobiotic detoxification by cytochrome P450, and neurodegenerative disease pathways (e.g., Alzheimer’s and Huntington’s diseases), implying its potential in metabolic detoxification and neuroprotection.

Additionally, the immune cell infiltration correlation matrix (Figure 7G) demonstrated significant associations between these signature genes and various immune cell subsets (e.g., memory B cells, activated dendritic cells, M1/M2 macrophages) at different significance levels (*p* < 0.05 to *p* < 0.001). Notably, *CCL2* and CD44 showed positive correlations with pro-inflammatory immune cells (e.g., activated NK cells, neutrophils), while *CALM3* and *JUN* exhibited negative correlations with some resting immune cells (e.g., resting mast cells). These correlations further echo the immune-regulatory and metabolic pathway enrichments identified by GSEA, suggesting that these exercise-related signature genes shape the pathological microenvironment of cocaine addiction by coordinating metabolic adaptation, immune responses, and cell signaling.

### 3.7. Directional Correlation Analysis Between Exercise and Addiction

**Figure 8:**
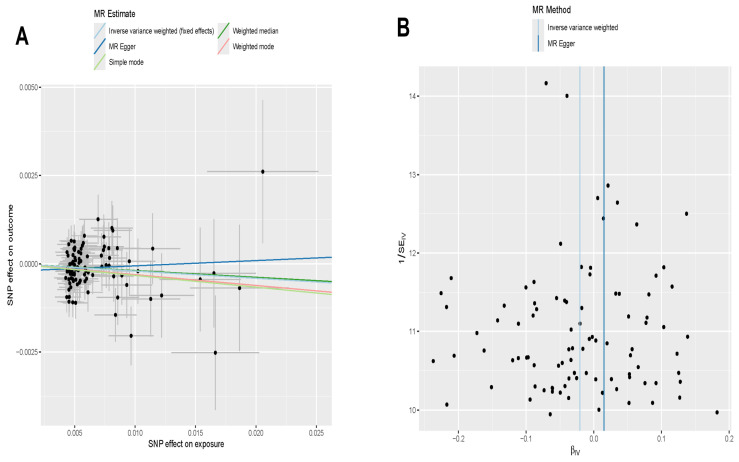

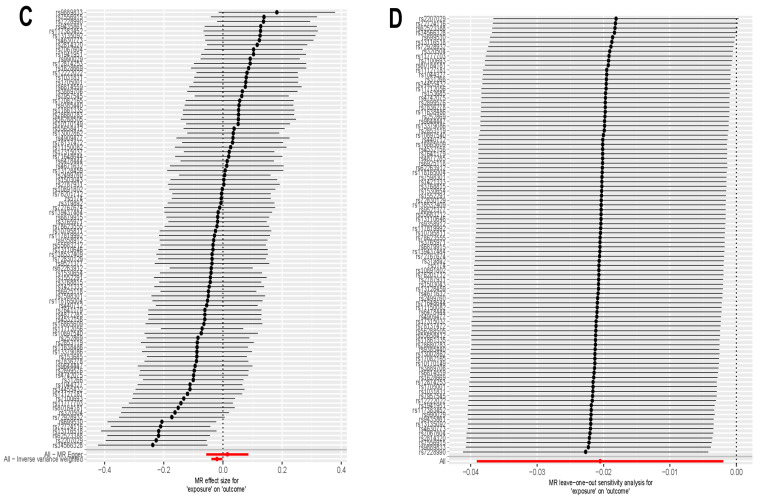
Mendelian randomization analysis of the direct correlation between exercise and addiction. (**A**) Causal effect of exercise on addiction, using ukb-B-4000 as the exposure and ukb-d-20552_2 as the outcome. (**B**) Causal effect of each SNP on addiction risk. (**C**) Overall heterogeneity of MR estimates for the impact of exercise on addiction. (**D**) Causal effect of exercise on addiction when individual SNPs are sequentially excluded.

To investigate the potential causal relationship between exercise and addiction, we performed two-sample Mendelian randomization (MR) analysis using exercise as the exposure, genetic variants (SNPs) as instrumental variables, and addiction as the outcome. Figure 8A illustrates the causal estimates obtained from five MR methods (inverse variance weighted (IVW), MR-Egger, weighted median, weighted mode and simple mode), with the IVW method indicating a significant negative association between genetically proxied higher levels of exercise and addiction risk (OR = 0.979, 95% CI: 0.965–0.997, *p* < 0.05). Although MR-Egger showed a similar direction of effect, the association did not reach statistical significance (*p* > 0.05). Cochran’s Q test did not reveal substantial heterogeneity among the instruments (Q = 75.56, *p* = 0.3642), and the MR-Egger intercept was close to zero (intercept = 0.0001, *p* = 0.3396) (Supplementary Table S6), suggesting no strong evidence of directional horizontal pleiotropy. Consistent with this, the MR-PRESSO global test did not detect significant outlier SNPs (*p* = 0.388). Figure 8B evaluates directional pleiotropy through SNP distribution, where symmetric clustering along both IVW and MR-Egger regression lines further supports the absence of systematic bias. Figure 8C (funnel plot) displays approximately symmetric SNP-specific effect estimates, in line with the non-significant Cochran’s Q statistic. Figure 8D (leave-one-out analysis) confirms result stability, as sequential exclusion of individual SNPs yielded consistent causal estimates. Collectively, these analyses support a potential causal protective effect of exercise against addiction within the MR framework.

## 4. Discussion

This study integrated transcriptomic profiling, bioinformatics, machine learning, and Mendelian randomization, and identified exercise-related signature genes potentially associated with cocaine addiction, thereby shedding new light on the underlying mechanisms linking exercise to cocaine addiction. Our analysis of the GSE54839 dataset identified 244 DEGs between cocaine-addicted and control groups, with 27 of these being exercise-related. These 27 genes represent the intersection between the 244 DEGs and a panel of 918 exercise-related genes retrieved from GeneCards, which includes classical exercise-responsive factors such as BDNF and IGF1. However, these canonical factors did not meet the predefined thresholds for differential expression in this midbrain dataset and were therefore not retained among the exercise-related DEGs. The top upregulated and downregulated genes, such as CCL2, JUN, PVALB, and TH, have been previously implicated in neurobiological processes relevant to addiction and exercise [25,26,27,28]. For instance, CCL2, a chemokine involved in immune cell recruitment, has been shown to play a role in neuroinflammation associated with drug addiction [25,29], while TH, which encodes tyrosine hydroxylase, is critical for dopamine synthesis, a neurotransmitter closely linked to cocaine’s rewarding effects [30]. The high reproducibility of these exercise-related DEGs in PCA supports their potential as exploratory biomarkers of cocaine addiction.

The construction of the PPI network revealed hub genes, including IL1B, IL6, and CCL2, which are key players in immune and inflammatory responses [31]. This aligns with functional enrichment analyses that highlighted immune-related pathways such as the IL-17 and TNF signaling pathways. These pathways have been increasingly recognized as important mediators of the neuroadaptations underlying addiction [32]. The strong positive correlations observed between genes like CCL2, CD44, and CXCL8 further suggest coordinated regulation of immune processes in cocaine addiction, which may be modulated by exercise.

Immunological characterization revealed a skewed immune profile in cocaine-addicted individuals, with increased pro-inflammatory cells such as M0 macrophages and neutrophils, and decreased immunosuppressive cells like Tregs. This is consistent with previous reports of neuroinflammation in substance use disorders [33,34]. The correlations between exercise-related DEGs and these immune cell subsets suggest that exercise may exert its effects, at least in part, through immunomodulation. For example, the positive correlations of CCL2 and CD44 with pro-inflammatory cells align with their known roles in promoting inflammation [31,35], while the negative correlations of CALM3 and JUN with resting immune cells hint at potential regulatory mechanisms.

The identification of six exercise-related signature genes (CALM3, CCL2, CD44, CLIC1, JUN, and VCAM1) with AUCs between 0.714 and 0.868 in this dataset indicates moderate discriminative ability at the single-gene level and suggests that these genes may be explored as candidate molecular markers in future studies. Notably, these AUC values fall within the range reported for other biomarker-based models in cocaine- and stimulant-related conditions, such as plasma inflammatory mediators distinguishing primary from cocaine-induced major depression (AUC 0.867–0.914), serum HSP70 for cocaine dependence (AUC 0.948) and peripheral exosomal miR-184-3p for methamphetamine use disorder (AUC 0.823–0.902), although the differences in clinical phenotype, tissue source and study design preclude direct quantitative comparison [36,37,38]. Beyond their performance as putative markers, these genes are involved in diverse processes including metabolism, cell signaling, and immune function, as indicated by GSEA. For example, CCL2 enrichment in cytokine signaling and VCAM1’s involvement in neurodegenerative pathways further connect these genes to the pathological processes of addiction [39,40].

Notably, our two-sample MR analysis suggested a statistically significant protective association between genetically proxied higher levels of exercise and addiction risk, supporting the potential of exercise as a preventive or adjunctive therapeutic intervention for cocaine addiction. This finding is consistent with previous observational and interventional studies suggesting beneficial effects of physical activity on craving, mood and relapse in substance use disorders [41,42]. However, MR inference depends on several core assumptions (relevance, independence and exclusion restriction), and violations of these assumptions, particularly horizontal pleiotropy, may bias effect estimates [43]. Although we applied multiple complementary MR methods and sensitivity analyses to evaluate heterogeneity and directional pleiotropy, residual pleiotropy and other sources of bias cannot be ruled out. Taken together, the MR analyses support a possible protective effect of exercise on cocaine addiction, but the evidence remains provisional and will need to be corroborated by larger, independent datasets and complementary study designs.

However, several limitations of the present study should be acknowledged. First, the analysis was based on a single transcriptomic dataset (GSE54839) with a relatively small sample size, which may limit the generalizability of our findings; replication in independent cohorts is necessary to confirm the identified exercise-related signature genes. Second, the cross-sectional nature of the gene expression data precludes definitive conclusions about the temporal and causal sequence between transcriptional changes, exercise exposure and cocaine addiction. Third, the functional roles of these signature genes in mediating the protective effect of exercise against cocaine addiction require further experimental validation, such as in vitro and in vivo studies. Fourth, immune cell composition was inferred from bulk midbrain transcriptomic data using CIBERSORT, which was originally developed on blood-derived expression profiles. Thus, the estimated immune cell fractions are best regarded as semi-quantitative indicators of immune-related transcriptional activity rather than precise measurements of true immune-cell infiltration and would benefit from confirmation by single-cell or tissue-level approaches. Fifth, exercise-related genes were defined solely on the basis of GeneCards annotations, which may not fully distinguish genes directly regulated by physical activity from those indirectly linked through stress or inflammatory processes, so the exercise-related DEGs identified here should be considered putative mediators of exercise effects. Sixth, although MR analysis can strengthen causal inference by using genetic variants as instrumental variables [44], it does not identify the specific biological pathways involved, and the mechanisms by which exercise exerts its protective effect through these signature genes remain to be elucidated.

In conclusion, our study identifies exercise-related signature genes associated with cocaine addiction, reveals their involvement in immune regulation, metabolic pathways, and cell signaling, and suggests a potential causal protective effect of exercise against addiction within the MR framework. These findings provide novel insights into the molecular mechanisms linking exercise to cocaine addiction and highlight potential therapeutic targets and exploratory molecular markers for cocaine addiction. Future studies focusing on the functional validation of these signature genes and the development of exercise-based interventions tailored to these molecular targets may advance the prevention and treatment of cocaine addiction.

## Figures and Tables

**Table 1:** Expression statistics for the 27 exercise-related DEGs in the GSE54839 datase.

Gene Symbol	logFC	Change	AveExpr	*t*	*p*. Value	adj. P.Val	B
PVALB	−0.9040713	Down	10.9340726	−4.117985	0.00011699	0.01874068	1.08834444
TH	−0.6655347	Down	13.6742803	−3.459475	0.00099407	0.04754572	−0.8169993
CKMT1B	−0.5378571	Down	9.42642172	−2.7112424	0.00869799	0.12918337	−2.7085261
ALDH1A1	−0.5257757	Down	13.2106844	−2.5519271	0.01323805	0.16193745	−3.0666404
GLS	−0.4003609	Down	11.7096645	−2.1415717	0.03623662	0.26471224	−3.9074687
NTS	−0.3989501	Down	7.53491886	−2.222223	0.02999247	0.24193402	−3.7518751
ACOT7	−0.392685	Down	11.0059734	−2.9431282	0.00459224	0.09452388	−2.1578242
CALM3	−0.3877455	Down	13.9020062	−3.1237466	0.00273296	0.07349115	−1.7059998
CBS	0.40987253	UP	9.82353997	3.24744315	0.00189576	0.06206236	−1.3856415
MCL1	0.43759294	UP	8.63790681	5.51343691	7.57 × 10^−7^	0.00218308	5.63078405
EDN1	0.44110921	UP	8.47453027	2.54220771	0.01357469	0.16452647	−3.0879343
PNP	0.44485248	UP	8.61166132	3.12815678	0.00269794	0.0732806	−1.6947275
CLIC1	0.45165804	UP	9.52609315	5.13521173	3.13 × 10^−6^	0.00371219	4.34961648
CD44	0.45530446	UP	9.96710693	4.43777402	3.88 × 10^−5^	0.01210736	2.07828665
IL1B	0.48791033	UP	7.7498335	2.31589396	0.02394641	0.21959765	−3.5651186
VCAM1	0.54219228	UP	8.73761938	5.52828584	7.16 × 10^−7^	0.00218308	5.68163473
IL6	0.55541545	UP	7.47143369	2.67218286	0.00965536	0.13676059	−2.7978899
S100A9	0.57873832	UP	7.81671457	3.01240385	0.00377152	0.08619948	−1.986824
HSPB1	0.59167695	UP	12.1919345	3.47923393	0.00093495	0.04626419	−0.7628044
CXCL8	0.77885885	UP	7.73964873	2.98477665	0.00408093	0.08967083	−2.0553665
S100A8	0.80605271	UP	8.18308567	2.7360823	0.00813521	0.12530676	−2.6511752
BAG3	0.816936	UP	10.9550748	3.85386002	0.00028235	0.02710923	0.30076053
JUN	0.84145631	UP	10.6533235	4.41005037	4.28 × 10^−5^	0.01216747	1.99101944
SERPINH1	0.84327308	UP	8.45667748	3.33088725	0.00147448	0.05604694	−1.1647255
HSPA1A	0.86890436	UP	12.028583	3.22907177	0.00200263	0.06350456	−1.4337653
CDKN1A	1.02758502	UP	8.71041932	4.81688536	1.00 × 10^−5^	0.00669562	3.29612798
CCL2	1.09239267	UP	8.6513175	5.01330203	4.90 × 10^−6^	0.00494924	3.94318854

## Data Availability

The data sets supporting the results of this article are available in the Gene Expression Omnibus repository: GSE54839 (https://www.ncbi.nlm.nih.gov/geo/, accessed on 2 July 2025).

## Supplementary Materials

The following supporting information can be downloaded at: https://www.mdpi.com/article/10.3390/genes16121414/s1, Figure S1: Correlation matrix of 22 distinct immune cell subtypes within cocaine-addicted DEGs; Table S1: The results of 244 cocaine addiction differential genes; Table S2: The results of 27 exercise-related differential genes; Table S3: Mendelian randomization machine output results; Table S4: Simulation-based post hoc power for differential expression analysis at n = 10 per group; Table S5: SNPs representing genetically predicted physical exercise; Table S6: Sensitivity analysis of the significant MR results.
